# Mechanisms of Variability Underlying Odor-Guided Locomotion

**DOI:** 10.3389/fnbeh.2022.871884

**Published:** 2022-05-04

**Authors:** Liangyu Tao, Vikas Bhandawat

**Affiliations:** School of Biomedical Engineering, Science and Health, Drexel University, Philadelphia, PA, United States

**Keywords:** *Drosophila*, odor-guided locomotion, variability, stochastic, circuit

## Abstract

Changes in locomotion mediated by odors (odor-guided locomotion) are an important mechanism by which animals discover resources important to their survival. Odor-guided locomotion, like most other behaviors, is highly variable. Variability in behavior can arise at many nodes along the circuit that performs sensorimotor transformation. We review these sources of variability in the context of the *Drosophila* olfactory system. While these sources of variability are important, using a model for locomotion, we show that another important contributor to behavioral variability is the stochastic nature of decision-making during locomotion as well as the persistence of these decisions: Flies choose the speed and curvature stochastically from a distribution and locomote with the same speed and curvature for extended periods. This stochasticity in locomotion will result in variability in behavior even if there is no noise in sensorimotor transformation. Overall, the noise in sensorimotor transformation is amplified by mechanisms of locomotion making odor-guided locomotion in flies highly variable.

## Introduction

Variability is a hallmark of behavior and is observed across timescales (Tinbergen, 1951). On long timescales, variability has been studied in the migratory behavior of birds; birds display inter-individual variability in migratory patterns, timing, and kinematics such as migratory speed (Potti, 1998; Trierweiler et al., 2014; Fraser et al., 2019; Phipps et al., 2019). On shorter timescales, many studies have looked at variability in movement kinetics, kinematics, and endpoints of reaching movements (Gordon et al., 1994; Messier and Kalaska, 1999; van Beers et al., 2004; Wu et al., 2014). Even when movement kinematics, such as walking speed, is constrained to a constant value, studies in humans have shown that there is variability in properties such as step length and width (Sekiya et al., 1997; Collins and Kuo, 2013).

**FIGURE 1:**
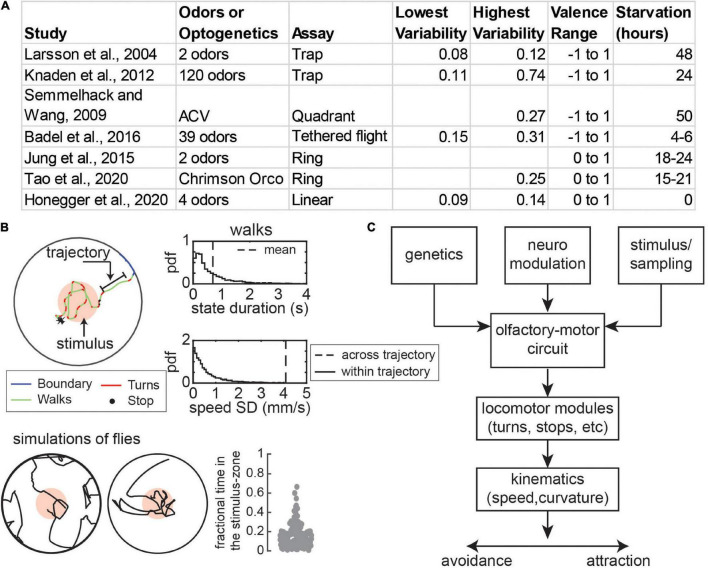
Persistence of locomotor states is an important contributor to variability in olfactory behavior. **(A)** Examples of variability in attraction to odors. Most sources of variability is measured as the standard deviation (SD) in attraction index with the exception of Knaden et al., 2012 and Jung et al., 2015, where it is represented by the interquartile range. **(B)** Top: In a circular arena with a concentric odor zone, fly locomotion can be represented as discrete states such as walks and turns (different colors) which last 700 millisecond on average (dotted line). During each state flies move with relatively stable speed and curvature as compared to across trajectories (characterized by the SD). Probability density distributions for durations and speed SD of walking trajectories are shown on the right. Bottom: This persistence leads to variability in sample trajectories. Over many samples, simulations of flies (*n* = 116) show a high a high degree of variability in the movement path and time spent in the odor zone (SD = 0.12). **(C)** Genetic factors, neuromodulation, and the dynamics of olfactory stimulus and sensorimotor sampling all can cause variability in the olfactory-motor circuit. This will result in variability in the performance of locomotor modules such as turns which results in variability in the time averaged attraction to odors. Panel **(B)** is adapted from Tao et al. (2020).

Given the ubiquity of behavioral variability, it is unsurprising that odor-guided locomotion in fruit flies or *Drosophila melanogaster* also shows variability. One large body of literature has focused on the idea of behavioral valence (attraction vs. repulsion) of flies to odors. Attraction or repulsion of a fly to an odor source is usually measured as the fraction of time a fly spends within an odorized region. These studies often utilize a wide array of odors and a wide range of behavioral assays ranging from a trap assay where a population of flies chooses between two odor traps to assays with a single fly in an arena with a single odorant zone (Figure 1A). Yet, regardless of the experimental setup or the odors used, there is a large variability in attraction (Figure 1A and methods) (Larsson et al., 2004; Semmelhack and Wang, 2009; Knaden et al., 2012; Jung et al., 2015; Badel et al., 2016; Honegger et al., 2020; Tao et al., 2020). As a simple illustration of the large variability, consider an experiment in which the standard deviation (SD) in attraction is 0.09 (Figure 1A), one of the lowest values in our survey of the literature. A SD of 0.09 with a mean attraction of 0.5 means that 95% (± 2 SD) of attraction would fall between 0.32 and 0.68, a large range.

Recently, research on odor-guided locomotion has moved past simple measures of valence to the moment-by-moment change in locomotion that accompanies attraction or repulsion. This advance parallels advances in ethological techniques to perform pose estimation (Mathis et al., 2018; Graving et al., 2019; Pereira et al., 2019), identification of behaviors (Dankert et al., 2009; Kabra et al., 2013; Berman et al., 2014; Wiltschko et al., 2015; Tao et al., 2019), and high throughput experimentation (Branson et al., 2009; Buchanan et al., 2015; Werkhoven et al., 2019). In the context of fly locomotion and how odors affect it, one insight from studying the detailed mechanism is that fly locomotion is comprised of sequences of discrete movement states, i.e., flies move at a surprisingly constant speed and curvature for extended periods before making sudden changes. This persistence means that instead of deciding on speed and curvature on every step, flies make decisions at the beginning of a “state” which can last several steps (hundreds of milliseconds). As we will discuss at length in this review, this persistence means that each decision will be important and small differences in choices will drive large variability in sensory experience and the spatial spread of a population of flies.

The effect of locomotor persistence on variability is well-described by a recent study that employed a hierarchical hidden Markov Model (HHMM) (Tao et al., 2019). The HHMM is an unsupervised method to infer states based on speed and curvature in an unbiased way. The authors found that flies use about ten states – each state defined by characteristic speed and curvature that does not change much during the state – to walk around a small circular arena. These states are persistent and last about a second, a time during which a fly takes 10 steps on average. Although each fly in the dataset could have its own set of states, a single set of states modeled all the flies. Since flies utilize a single set of state, flies likely utilize the same building blocks during locomotion. These building blocks account for locomotion both before the odor was turned on and during the odor period (Tao et al., 2019). Although flies use the same states, there is large fly-to-fly variability in the time spent performing each state both in the absence and presence of odors. The variability in state usage results in behavioral variability since there is a large difference in speed and curvature between states. In contrast to between states, this model shows a tight distribution of kinematics within a state, implying that flies maintain consistent kinematics (speed and curvature) for about a second – a time during which the fly takes ∼10 steps. Qualitatively, these states represent characterizations of different types of walking, stopping, and turning states.

The HHMM model shows that locomotion consists of persistent states where each state represents different types of walking, stopping, and turning states. The insights from the HHMM model – that persistence of a state can cause variability – can also be captured by a much simpler model with four states – walk, stop, turn, and boundary (Tao et al., 2020; Figure 1B). Each transition into a given state is well-described by the average kinematics (e.g., speed), but different transitions can have widely different speeds. The persistence is shown by the fact that states last on average 700 milliseconds within which the variation in speed is much less than the variation observed across states (Figure 1B). The result of this variation is that the tracks of the fly and attraction to odors are highly variable even though each fly is executing the same algorithm (Figure 1B).

Both the variability in olfactory behavior (Figure 1A) and the role of the nature of locomotion itself in creating this variability (Figure 1B) has not been systematically explored. Here, we will review potential mechanisms behind variability in odor-guided locomotion. At any moment a given fly has a given locomotor or search algorithm which is determined by its sensory environment and its state acting on its locomotor circuits. Odors affect attraction and repulsion by changing how these different locomotor states are used, and how different locomotor variables such as speed and curvature are chosen in a given state (Figure 1C). Thus, variability in olfactory behavior can result from differences in sensorimotor transformations which in turn can result from irreversible genetic differences, from reversible neuromodulatory differences, or from sampling noise. We will draw on work aimed at understanding both variability in odor valence and odor-driven locomotion. We will emphasize that the noise in sensorimotor transformations when coupled with persistence in locomotion can be an important source of variability in genetically identical flies. The review is organized into four main sections. In the first section, we will orient the reader on the structure and function of the fly’s olfactory system. In the remaining three sections, we will discuss variability arising from genetic differences, neuromodulation with an emphasis on hunger, and from sampling noise in turn.

## Signal Processing in the *Drosophila* Olfactory Circuit

**FIGURE 2:**
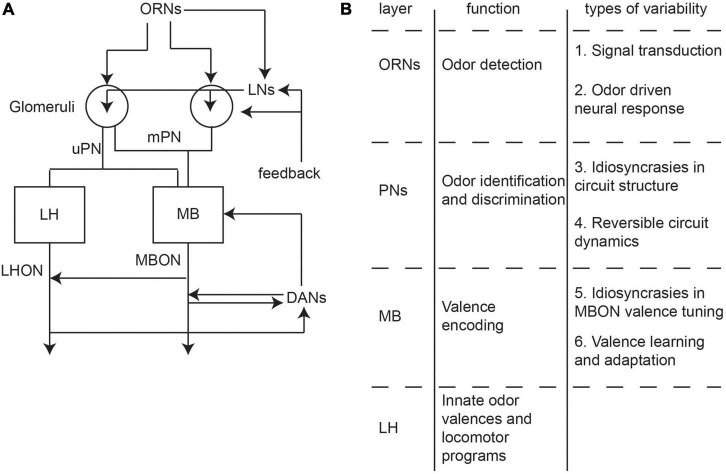
Information flow and properties of the *Drosophila* olfactory circuit. **(A)** Information flow of the fly olfactory circuit. First order olfactory receptor neurons (ORNs) detect odors and synapse with uniglomerular and multiglomerular projection neurons (uPNs and mPNs, respectively) in the glomeruli. Local neurons (LNs) provide lateral connections. PNs synpase into the mushroom body MB and lateral horn (LH), which act as third order processing centers. Dopaminergic neurons modulate MB activity. Mushroom body output neurons (MBONs) and lateral horn output neurons (LHONs) carry information into higher order circuits. **(B)** A table of the main function at each layer of the olfactory circuit as well as where variability will arise.

Olfactory processing in *Drosophila* can be broken down into three layers of processing (Figure 2A). First, odors are detected by the receptors of ∼1,400 olfactory receptor neurons (ORNs) located in the antennae and maxillary palps. These olfactory organs have hair-like protrusions that each house the dendrites of one to four ORNs (Vosshall et al., 1999). ORNs can be segregated into distinct classes based on the expression of 51 receptor types (de Bruyne et al., 1999, 2001; Bates et al., 2020). At the signal detection level, odorant-binding proteins (OBPs) facilitate the transport of odorants to bind with olfactory receptors (ORs). Beyond OBPs and ORs within a single sensillum, ORN signal transduction will be influenced by sensillar morphology, lymph fluid biochemistry, and physiological crosstalk between sensillar cells (Schmidt and Benton, 2020).

Olfactory signal transduction will ultimately lead to ORN spiking activity. The rate of spiking increases immediately following odor onset, then adapts to a stable but elevated level. The level of activation for each class of ORN is dependent on the odorant, and also has a non-linear dependence on its concentration within the odor plume (Hallem et al., 2004; Hallem and Carlson, 2006). The relationship between odor concentration and ORN spiking response also depends on stimulus history (Nagel and Wilson, 2011; Martelli and Fiala, 2019). At odor offset, the neural activity of many types of ORNs is inhibited for an extended period that can last for upwards of a few seconds.

The ORNs project to 51 glomeruli in the antennal lobe where they synapse with second-order projection neurons (PNs) which carry information into higher-order olfactory processing centers (Bates et al., 2020). PNs can be classified into uniglomerular PNs (uPNs) that receive input from a single glomerulus and multiglomerular PNs (mPNs) that receive input from multiple glomeruli (Figure 2A; Bates et al., 2020). In addition to the PNs, local neurons (LNs) connect multiple glomeruli within the antennal lobe through lateral connections (Figure 2A). The computation in the antennal lobe results in an increase in the separability in odor representations and a decrease in variability in response to a given ORN class (Bhandawat et al., 2007; Olsen et al., 2010; Wilson, 2013).

From the PNs, olfactory information is next transmitted to third-order processing centers called the mushroom body (MB) and the lateral horn (LH) (Figure 2A). In the MB, PNs form random synapses with on average 7 Kenyon cells (KC) in the MB calyx (Jefferis et al., 2007; Butcher et al., 2012; Caron et al., 2013). The output of the MB calyx converges into a small set of 34 output neurons called mushroom body output neurons (MBONs) that are separated into 15 different compartments (Tanaka et al., 2008; Aso et al., 2014b; Bates et al., 2020). Functional studies have shown that the MBON activity patterns likely encode the valence of an odor. This valence can be remapped or learned through synaptic plasticity brought about by dopaminergic neurons (DANs) that enervate each compartment of the MBONs (Aso et al., 2014b). DANs in turn can receive inputs from both the MBONs as well as input from the lateral horn output neurons (LHONs) (Dolan et al., 2019; Li et al., 2020).

The LH is comprised of local neurons and output neurons. These neurons receive excitatory input from both the uPNs and mPNs as well as inhibitory input from the mPNs (Bates et al., 2020). The LHONs and MBONs project downstream into multiple fourth-order processing centers. The MB and the LH are highly interconnected via both direct connections (Aso et al., 2014b; Dolan et al., 2019) as well as via recurrent connections from MBONs to PN axons in the LH (Bates et al., 2020). The specific function of the LH is currently being actively investigated, but specific classes of neurons have been shown to drive innate odor valence as well as specific locomotor programs such as turning or wingbeat frequency during flight (Dolan et al., 2019; Varela et al., 2019).

The MB and LH represent what is the final stage of the relatively stereotyped olfactory circuit. From here olfactory information form multiple convergent and divergent pathways to higher order circuits as well as recurrent pathways to the aforementioned layers of olfactory processing neurons (Bates et al., 2020; Scheffer et al., 2020; Scaplen et al., 2021). Recent studies have shown that these higher order circuits, especially those in the central brain allow flies to integrate and switch between multisensory information such as wind and visual cues during odor guided locomotion to generate a representation of the direction of the olfactory source (Suver et al., 2019; Okubo et al., 2020; Matheson et al., 2021). Variations in circuit activity at the level of the central complex may ultimately explain variability in movement reorientation when the fly is turning during olfactory guided locomotion. The role of central complex in odor-guided locomotion is discussed in detail in other recent reviews (Hulse et al., 2021; Fisher, 2022).

## Genetics as a Source of Variability in *Drosophila* Odor-Guided Locomotion

At each step described above, variability can arise from genetic differences which can affect different aspects of the sensorimotor transformation as reviewed below. First, subtle changes in genes that are directly involved in various aspects of olfactory processing can affect sensorimotor transformation. There is a growing body of evidence particularly at the level of ORNs that supports contribution due to this mechanism. Even in isogenic flies, accumulations of polymorphisms can lead to behavioral variability (Mollá-Albaladejo and Sánchez-Alcañiz, 2021). For instance, naturally occurring single nucleotide polymorphism (SNP) in OBPs 99a-d has been shown to contribute to the phenotypic variability in the aversion to benzaldehyde (Wang et al., 2007). The authors found in a follow-up study that SNPs in different OBPs in the 99a-d complex can have a varied effect on olfactory behaviors (Wang et al., 2010). Similarly, natural polymorphisms in multiple ORs have been found to have a significant association with variations in odorant-specific valence (Rollmann et al., 2010; Richgels and Rollmann, 2012).

Single nucleotide polymorphisms can also affect olfactory behavior via network pathways involved in olfactory signal transduction, neurogenesis, and neural connectivity (Figure 2B; Swarup et al., 2013; Arya et al., 2015). A recent study provides evidence that genetic variation in the Or22 locus leads to significant differences in the functional neural response properties of its corresponding class of ORN, which in turn correlates with a preference for ethyl hexanoate, an odor that strongly stimulates this ORN (Shaw et al., 2019, 2021).

In addition to single-neuron effects, individuality in the genetic code can lead to wiring and structural variability in neural circuits (Figure 2B). A recent study looking at a large population of inbred flies over 9 different behavioral assays showed that individual differences in genes related to development (e.g., Hedgehog signaling, Wnt signaling) and neural function (e.g., vesicle release) may be involved with behavioral variability (Werkhoven et al., 2021). This study also implicated genes involved in cellular respiration and protein translation in behavioral variability.

Despite recent efforts, the mechanistic effect of variability of most genes on animal-by-animal variability in odor guided locomotion is still unknown. These effects may present themselves through careful anatomical and functional studies. In the antennal lobe, electron microscopy studies show that the connectivity from ORN to PN are variable. In one study, the authors found that there is a high degree of synaptic variability, which leads to the contamination of ORN spike count information (Tobin et al., 2017). Some variability in this connectivity will be compensated for. For instance, one hemisphere may have smaller PN dendritic sizes but compensate with more synapses to generate similar postsynaptic membrane potential responses to pre-synaptic ORN input. In addition to the ORN to PN connections, LNs have also been found to exhibit variability in fine-scale connectivity patterns which undergo both developmental and experience-dependent plasticity (Chou et al., 2010). However, the extent to which this variability leads to variability in sensory processing and ultimately behavioral variability is unclear.

Finally, an important mechanism for genetic variability is the plasticity effect of different genes that alter olfactory valence (Figure 2B). In the MB, there are many genes shown to be important for olfactory memory (Kahsai and Zars, 2011). It has been shown that while the tuning of individual MBON compartments is the same across hemispheres of an individual fly, the tuning of these compartments is different across animals. This source of individuality is linked to the rutabaga (*rut*) gene (Hige et al., 2015). In the MB, both the *rut* and *dunce* gene are involved in the synthesis and degradation of cAMP, and mutations in these genes have been shown to affect signal transduction (Renger et al., 2000).

While these studies show that genetic variability can lead to individuality through potential changes in signal transduction and circuit wiring, they will not be the only source of this variability. For example, a recent study in the fly visual system showed that left/right wiring asymmetry for a set of neurons called the dorsal cluster neurons is caused by stochastic wiring during development and not genetic differences. The extent of the wiring asymmetry explains the ability of individual flies to orient toward a visual object (Linneweber Gerit et al., 2020).

## Neuromodulation May Drive Shifts in Valence Through Changing Excitatory-Inhibitory Balance

A second mechanism for variability is through internal states such as hunger which have been shown to drastically alter the behavioral valence of odors through neuromodulation (Figure 2B). In the antennal lobe, such neuromodulators act upon both the LN and uPN to generate variability in attraction to odors. In a recent study, it was found that feeding flies a serotonin synthesis inhibitor (alpha-methyltryptophan) or expressing a mutant allele of the dopamine receptor gene (Dop1R1) resulted in a decrease in the variability of odor preference. Meanwhile, feeding flies a dopamine precursor (L-DOPA) increased odor preference variability (Honegger et al., 2020).

**FIGURE 3:**
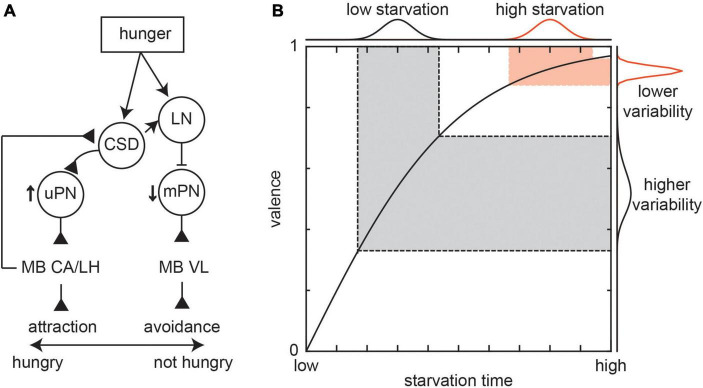
Effect of internal states on behavioral variability. **(A)** Effect of hunger on larvae attraction or avoidance to geranyl acetate. When hungry, the CSD neuron potentiate attraction mediating uPN responses while LNs inhibit aversion mediating mPN responses through glutamatergic mPNs. This leads to a switch from avoidance to attraction through downstream connections to the mushroom body calyx (MB CA), mushroom body vertical lobe (MB VL), and lateral horn (LH). Figure based on Vogt et al., 2021. **(B)** The variability in behaviors such as attraction depends on the relationship between the behavior and internal states like hunger (represented by starvation time). In this cartoon, two groups of flies that have the same variance in starvation times, the flies that are starved more should show less variability in valence. However, experiments typically show a higher level of valence variance than that predicted by theoretical average relationship curves.

The effect of serotonin on the antennal lobe neurons is likely a result of action of a well-studied group of serotonergic neurons, that modulate both LN and PN activity, called the contralaterally projecting serotonin-immunoreactive deuterocerebral (CSD) neurons (Zhang and Gaudry, 2016). These neurons are conserved among multiple insect taxa (Kent et al., 1987; Python and Stocker, 2002; Dacks et al., 2006). Interestingly, it was found that thermogenetic activation of the CSD neurons did not change the attraction to or variability in the attraction to the odors (Honegger et al., 2020). However, a recent paper in larvae showed that CSD neurons are necessary for hunger-driven changes in olfactory behavior. When satiated, larvae avoid geranyl acetate; when hungry, CSD neurons cause an increase in attraction to geranyl acetate by directly potentiating attraction mediating uPN responses while indirectly inhibiting aversion mediating mPN responses (Figure 3A; Vogt et al., 2021). The circuit motif of hunger promoting activity in attraction mediating neurons and reducing activity in aversion mediating neurons appears in both the antennal lobe (Root et al., 2011; Ko et al., 2015) and mushroom body (MB) (Tsao et al., 2018).

Since the level of hunger can play a key role in behavioral variability, most laboratory studies control hunger though controlling starvation time. In the antennal lobe, the duration of starvation leads to a negative exponential change in PN activity (Root et al., 2011). In the same study, it was shown that the mean time spent finding food follows a similar pattern. Such a mechanism suggests that changes in valence caused by variability in hunger levels should be less at large starvation values (Figure 3B). However, most studies show that even after long periods (24+ h) of starvation, there is still a high degree of valence variability (Figure 1A). In such scenarios, variability can still arise from neuromodulation. One potential explanation is because while the average effect of hunger on neural activity across individuals and trials saturates after long starvation periods, there is still variability in neural activity around the average that can reflect variability in activity in the antennal lobe, the effect of other sensory and higher order circuits that input into the antennal lobe, and variability in the amount of neuromodulation. Furthermore, while we have highlighted one potential mechanism of hunger, this state affects behaviors through a multitude of parallel mechanisms. For instance, this variability may reflect an increase in exploratory drive in a bid find the food source. This process is driven by a metabolic pathway where starvation drives an increase in the adipokinetic hormone, which in turn drives octopaminergic cells to promote foraging associated hyperactivity (Yang et al., 2015; Yu et al., 2016).

While hunger is the most well studied and one of the most important internal states for odor guided locomotion (especially in the context of food odors), there are many other internal states that can affect odor guided locomotion. For instance, the nutritional and social history of flies can affect both olfactory driven locomotion and attraction to specific odors (Lebreton et al., 2014; Jung et al., 2018; Huang et al., 2020). Finally, beyond internal states, trial-by-trial variability may arise from differences in the behavioral state of the fly. For instance, flies are attracted to CO_2_ when in an active foraging state but avoid CO_2_ when moving at a slower speed (van Breugel et al., 2018). The internal states and mechanisms described here exemplify a wider range of processes; some of these processes are detailed in other recent review (Grunwald Kadow, 2019; Lin et al., 2019; Maloney, 2021; Devineni and Scaplen, 2022).

## Variability in Sensorimotor Transformation Is Amplified by Stochastic and Persistent Behavioral Choices

In nature, flies will often navigate complex landscapes involving multiple odor sources where rather than a continuous odor gradient, flies experience odors as pulses – odor plumes – resulting from turbulent winds (Crimaldi and Koseff, 2001; Celani et al., 2014). To navigate these environments, the *Drosophila* will either fly or walk as it approaches the odors. There will be variability in sensorimotor transformations underlying the navigational strategies during each phase. Here, we will focus on the walking phase of odor guided locomotion.

**FIGURE 4:**
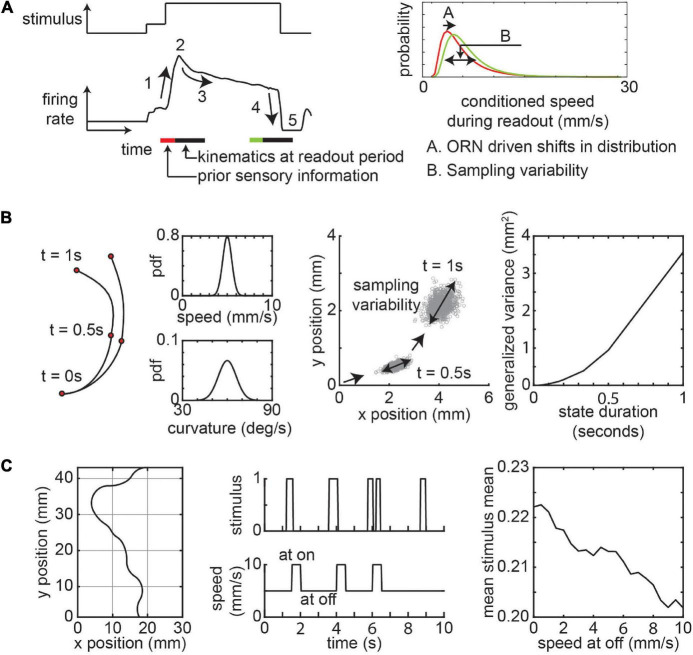
Variability due to sensorimotor transformations and sampling. **(A)** A schematic of odor stimulus and ORN response. The response is characterized by a rising edge (1), peak response (2), adaptation (3), falling edge (4), and inhibition (5). The speed bouts of curved walks (readout period) conditioned on ORN activity follows a lognormal distribution. The distribution changes based on ORN activity. **(B)** Variability in sensorimotor transformation will result in sampling variability. Left: Toy example of two consecutive instances of curved walk with constant speed and curvature sampled from normal distributions. Middle: Positions from 1,000 simulations starting at position (0,0) facing in the positive × position with a trajectory persistence of 0.5 s show variability increases with consecutive samples. Right: The generalized variance in positions after 5 s increase with increasing state persistence. **(C)** Effect of locomotor strategy on sensory experience. Left: Sample 10 s trajectory of a fly moving through an environment with constant average stimulus intensity, but with variable frequencies at each spatial block (bounded by gray). Middle: Stimulus experienced by the fly during the period as it chooses a lower speed when it experiences no odors. Right: The mean of the mean stimulus experienced by simulations of flies as a function of off speed (*n* = 5,000/speed at off). See methods for further details about simulations in **(B,C)**.

Far from the odor source, the frequency of plume encounters is small. A fly will encounter a pulse of odor such as the one shown in Figure 4A (from an actual experiment) and respond with the corresponding ORN activity (Figure 4A). The behavioral variability comes from two sources. First, odorant history and differences in ORN activity experienced by flies across separate odor encounters will lead to changes in the average locomotor kinematics such as speed and curvature (Figure 4A). Studies in wind tunnels show that the temporal dynamics of these sensorimotor transformations is complex and dependent on odor concentration and wind (Álvarez-Salvado et al., 2018; Demir et al., 2020). Recently studies have used open loop optogenetics to dissect the individual effects of ORN activation. A recent study using optogenetics show that even trial-by-trial differences in locomotion when crossing a static stimulus zone can lead to differential ORN activity (Tao et al., 2022).

A second source of noise is the stochasticity in locomotor kinematics across locomotor state transitions and the decision to transition between states. While the average sensorimotor transformation can be predicted from ORN activity in studies where other external factors like wind is controlled, there will be a high level of variability around this average. As such one way to think about this is that given the same olfactory stimulus information, flies will modulate their future locomotion by sampling from a probabilistic distribution (Figure 4A). The properties of this distribution (such as mean and variance) may be estimated by past ORN experience (Tao et al., 2022). If flies continuously update their speed and curvature on a moment-by-moment basis, then the positional variability due to sampling noise will be small. However, the variability arising from sampling noise is magnified when flies maintain relatively consistent kinematics for long (hundreds of milliseconds to seconds, Figure 1B) periods. This can be shown using a simple agent-based simulation where the agent moves at a constant speed and curvature based on samples from a gaussian distribution at fixed time intervals (Figure 4B and methods). The resultant spread of the flies in space increases as the interval between samples increases (Figure 4B). This means that two flies starting at the same position in space experiencing similar odor stimulus will have divergent positions and paths at the end of an instance of a locomotor state. In a spatially inhomogeneous odorant environment, this spatial dispersion in positions will have knock-on effects as the sensory experience of different flies diverge leading to greater variability in behavior.

In addition to locomotor kinematics, decisions to transition between walking, turning, and stopped states have been shown to be stochastic. How flies implement these decisions is dependent on the type of decision as well as the environment that the fly is locomoting in. For instance, flies implement stochastic sequential integration of odor plume encounters in transitioning from stops to walks and use the timing of odor encounters to modulate the transition from walks to stops (Demir et al., 2020). Furthermore, flies can bias their upwind turning based on the combination of the frequency and the intermittency of odor encounter (Álvarez-Salvado et al., 2018; Demir et al., 2020; Jayaram et al., 2022).

As the fly moves closer to the odor source, the frequency of odor encounters will increase. Effects discussed above will be further exacerbated as frequent odor encounters will drive history dependent ORN firing rate adaptation which creates a potential for greater variety in possible responses. Consider a temporally changing olfactory environment where the mean and variance of the stimulus is spatially conserved, if flies adopt a simple strategy of slowing down when not experiencing an odor plume, the mean in odor experience will increase (Figure 4C and methods). This increase in mean odor experience will depend on how much the fly decreases its speed. The gain in the ORN dose-response curve decreases with an increase in stimulus mean and variance (Gorur-Shandilya et al., 2017). At the population level, the sensitivity to odorant concentrations follows a power-law distribution and this response sensitivity adapts to stimulus intensity (Si et al., 2019). This means that flies can experience vastly different sensory input based on both statistics of the odorant environment and how the fly chooses to locomote within the environment.

In addition to the effect of recent sensory experience in driving behavioral variability, the sensorimotor transformations also exhibit adaptations over the course of tens of seconds to minutes. In a static odor landscape, the timescale of this adaptation coincides with changes in the attraction index (Tao et al., 2022). This adaptation likely reflects a longer timescale change in the perception of the odor based on the motivation of the fly to continue the search for the odor. A recent study showed that there is a large variability in the distance flies traveled on food patches before deciding to give up (van Breugel, 2021). Using an agent-based model of variable decision making, the author showed that this variability may enhance the metabolic efficiency in finding the food source. In the MB, DANs modulate MBON neurons and induce plasticity of KC to MBON connections to cause changes in odor valence (Aso et al., 2014a). The output of MBONs makes many connections with the LH, which is thought to drive innate behaviors and different motor programs (Dolan et al., 2019). This suggests that the longer timescale adaptations in locomotion and valence can be driven by the MB. This process, which depends on each flies’ experience and internal states may be a potential way to explain the variability in longer timescale odor valence and locomotion (Grunwald Kadow, 2019).

## Conclusion

Behavioral variability is a central feature of natural behaviors. Odor-guided locomotion performed by *Drosophila* is a key model system to study principles and sources of behavioral variability. Traditionally, variability is commonly attributed to genetic and neuromodulatory factors. Indeed, even in isogenous populations, small amounts of genetic variability may cause variability in phenotype expression. Such a process may allow a population of animals to limit the risk of going extinct in an expectedly ever-changing environment. Meanwhile, neuromodulation allows animals to flexibly control their behaviors in response to their internal needs or wants. But beyond these factors, another less discussed source of variability arises from stochasticity of behavioral choices and their persistence. Over multiple rounds of decision, this source of variability will drive noticeable variability in attraction and spatial position across a population of flies.

The presence of persistent locomotion is a ubiquitous feature of locomotion ranging from sharks to *Drosophila* to humans (Reynolds and Frye, 2007; Humphries et al., 2010; Rhee et al., 2011). This feature is predicted to provide ethological benefits in many environments by multiple theoretical frameworks for animal search ranging from Lévy flights to infotaxis. For instance, the power-law distribution of trajectory persistence during Lévy walks, although controversial, is predicted to be optimal in environments with random and sparse odor sources (Viswanathan et al., 1999). Meanwhile, infotaxis predicts long persistent path trajectories far from an odor source that shorten in duration in regions with high odor information accumulation (Vergassola et al., 2007). While potentially suboptimal, the infotaxis framework allows animals to reliably locate an odor source (Loisy and Eloy, 2021). But beyond potential ethological benefits of long persistence trajectories, there is a growing source of literature that shows how these frameworks that generate long persistence trajectories can arise naturally from biomechanical mechanisms of locomotion and neural mechanisms of decision making (Calhoun et al., 2014; Reynolds, 2015, 2021; Abe Masato, 2020).

Meanwhile, the presence of noisy sensorimotor transformations can arise from a multitude of factors. First, genetic, biomechanical, metabolic, and history-dependent experiences can influence idiosyncratic differences in sensorimotor transformations. Second, internal and external behavioral states can influence locomotor transformations across sensory experience. Finally, there will be natural, uncontrollable variations in locomotor speed and curvature likely arising from motor noise or various sources of noise in the brain (Faisal et al., 2008). Even in highly practiced tasks such as arm reaching, small variations in neuronal activity in the premotor cortex of monkeys has been shown to drive trial-by-trial movement variability (Churchland et al., 2006). During odor-guided locomotion where the goal of the animal is not to control the kinematics of locomotion explicitly and precisely, these sources of noise in locomotor kinematics will be larger. But beyond the biological origins of movement variability, this variability can be ethologically beneficial as a lack of movement variability can result in rigid locomotor search patterns that limit the ability of an animal to effectively search for resources.

## Materials and Methods

### Data Curation

Standard deviations (SD) reported in Figure 1A were obtained from the relevant articles through the raw data when available or through estimation of error bounds using WebPlotDigitizer (Rohatgi, 2021). As most studies report the standard error of the mean (SEM), the SD was calculated by multiplying the SEM by the square root of the reported sample size. For papers with box plots, WebPlotDigitizer was used to obtain the interquartile range. Below is a table of the relevant figures that error bounds were reported from, and the method used.

**Table:** 

	Figure number	Method
Larsson et al., 2004	Figure 7	WebPlotDigitizer
Knaden et al., 2012	Figure 1	WebPlotDigitizer
Semmelhack and Wang, 2009	Figure 2	WebPlotDigitizer
Badel et al., 2016	Figure 1	WebPlotDigitizer
Jung et al., 2015	Figure 3	WebPlotDigitizer
Tao et al., 2020	Figure 1	Data
Honegger et al., 2020	Figure 1	Data

### Agent Model of Sampling Noise Variability

The speed was sampled from a normal distribution with a mean of 5 mm/s and an SD of 0.5 mm/s. The curvature was sampled from a normal distribution with a mean of 60 degrees/s and an SD of 3 degrees/s. For each simulation the duration of a trajectory is fixed, and the sampling rate was set to 30 Hz. A 1,000 agents were initialized at the origin (*x* = 0 mm, *y* = 0 mm, and an orientation θ = 0 degrees). At the start of each trajectory, each agent selects from the speed and curvature distribution. The position of each agent was then updated as follows:


(1) $$\theta \,\left(t\right)=\theta \,\left(t-1\right)+\frac{k\,\left(t-1\right)+k\,\left(t\right)}{2}$$

(2) x⁢(t)=x⁢(t-1)+s⁢(t)*cos⁡(θ⁢(t))

(3) y⁢(t)=y⁢(t-1)+s⁢(t)*sin⁡(θ⁢(t))

Where *k* is the sampled curvature and *s* is the sampled speed. After the agent has moved for the set duration, the agent initiates another trajectory by resampling from the speed and curvature distribution. This process repeats until a time of 5 s has passed.

The spread of agents at the end of the 5 s period can be approximated by a bivariate Gaussian distribution. These end positions were fit to a bivariate gaussian density function using MATLAB. The spread of this distribution was characterized by the generalized variance:


G⁢V=d⁢e⁢t⁢(Σ)

Where Σ is the covariance matrix.

### Agent Model of Locomotion Induced Changes in Sensory Input

To simulate a dynamically changing environment with conserved stimulus properties, we first segmented the odor space into grids of 10 mm by 10 mm. The temporal pattern of odor stimulus in each grid is modeled as a square wave with a 20% duty cycle and variable frequency sampled from a gaussian distribution centered around 0.5 Hz with a standard deviation of 0.1 Hz.

A 5,000 agents were initialized at the origin (*x* = 0 mm, *y* = 0 mm, and an orientation θ = 0 degrees). Each agent is set to move in trajectories lasting 0.5 s. At the end of each trajectory, the agent update its speed based on its latest sensory experience. If the agent is in an odor plume (stimulus = 1) at the time of trajectory transition, the agent will initiate a trajectory with a speed of 10 mm/s (On stimulus speed) and a curvature of 60 degrees/s. If the agent is instead not in an odor plume (stimulus = 0), then the agent will initiate a trajectory with a speed slower than or equal to 10 mm/s (Off stimulus speed) and a curvature of 60 degrees/s. The direction of curvature is random (50/50 left vs. right). The position of each agent is updated as described in equations 1 to 3. For each agent, we calculated the mean in stimulus over 2 min. Figure 4C2 shows the mean of the stimulus mean over all agents.

## Author Contributions

Both authors listed have made a substantial, direct, and intellectual contribution to the work, and approved it for publication.

## Conflict of Interest

The authors declare that the research was conducted in the absence of any commercial or financial relationships that could be construed as a potential conflict of interest.

## Publisher’s Note

All claims expressed in this article are solely those of the authors and do not necessarily represent those of their affiliated organizations, or those of the publisher, the editors and the reviewers. Any product that may be evaluated in this article, or claim that may be made by its manufacturer, is not guaranteed or endorsed by the publisher.
